# Benefit-to-harm ratio and cost-effectiveness of government-recommended gastric cancer screening in China: A modeling study

**DOI:** 10.3389/fpubh.2022.955120

**Published:** 2022-08-10

**Authors:** Shuxia Qin, Xuehong Wang, Sini Li, Chongqing Tan, Xiaohui Zeng, Meiyu Wu, Ye Peng, Liting Wang, Xiaomin Wan

**Affiliations:** ^1^Department of Pharmacy, The Second Xiangya Hospital, Central South University, Changsha, China; ^2^Department of Gastroenterology, The Second Xiangya Hospital, Central South University, Changsha, China; ^3^Xiangya Nursing School, Central South University, Changsha, China; ^4^School of Health and Related Research, Faculty of Medicine, Dentistry and Health, University of Sheffield, Sheffield, United Kingdom; ^5^PET-CT Center, The Second Xiangya Hospital, Central South University, Changsha, China

**Keywords:** gastric cancer, screening, endoscopy, cost-effectiveness, modeling, benefit-to-harm ratio

## Abstract

**Objective:**

Current guidelines recommend the gastric cancer risk score scale (GCRSS) for screening in gastric cancer (GC) high-risk populations in China. This study aimed to estimate the clinical benefits, harms, cost, and cost-effectiveness of the GCRSS screening strategy from a Chinese healthcare system perspective.

**Materials and methods:**

Using a microsimulation model, we evaluated 7 screening scenarios of the GCRSS with varying starting ages. We simulated 100,000 individuals from the age of 20 for each screening scenario. The main outcomes included GC incidence reduction, number of cause-specific deaths, costs, quality-adjusted life year (QALY), incremental cost-effectiveness ratio (ICER), and benefit-to-harm ratio. Deterministic and probabilistic sensitivity analyses were done to explore the robustness of model findings.

**Results:**

Screening with the GCRSS strategy at the age of 40 years (40-GCRSS) provided the greatest reduction of GC incidence by 70.6%, with 7,374 GC deaths averted per 100,000 individuals and the lowest benefit-to-harm ratio of 0.392. Compared with no screening or previous less costly strategy, at a willingness-to-pay (WTP) threshold of $37,655 per QALY, the 40-GCRSS strategy was cost-effective, with ICERs of $12,586 and $29,115 per QALY, respectively. Results were robust across univariate and probabilistic sensitivity analyses. The 40-GCRSS strategy showed a 0.856 probability of being cost-effective at a $37,655 per QALY WTP threshold.

**Conclusions:**

The findings suggest that the GCRSS strategy is effective and cost-effective in reducing the GC disease burden in China from a Chinese healthcare system perspective. Screening from the age of 40 would be the optimal strategy.

## Introduction

Gastric cancer (GC) remains a common gastrointestinal tumor in China, threatening human health (1). There are over 670 thousand new diagnosed cases and about 500 thousand dead cases every year in China, which accounts for around 42 and 45% of the world, respectively (2, 3). At present, reducing the morbidity and mortality of GC is a major public health problem that needs to be solved (4, 5).

The prognosis of GC depends on the timing of detection and treatment, and prognosis of the advanced-stage detection is poor. In China, nearly 90% of GC patients were detected at an advanced stage (6). Although the endoscopic examination is an effective GC screening approach, even in developed countries with a high incidence rate of GC, such as Japan and Korea, it is impossible to screen GC for the whole population (7, 8). Only screening high-risk individuals of GC may be an effective and feasible method to reduce the disease burden of GC in China.

According to China's guidelines for diagnosis and treatment of GC, *Helicobacter pylori* (Hp) infection and gastric mucosal atrophy are important factors for identifying GC high-risk groups (9). Hp was classified as a class I carcinogen of GC by the International Agency for Research on Cancer as early as 1994 (10), which is the principal etiologic factor for the development of non-cardia GC, with an estimate of 75% of all the non-cardia GCs related to Hp infection (11, 12). Fortunately, Hp is considered to be a controllable environmental factor (13), and its eradication would effectively reduce the incidence of GC (14). Gastric atrophy and intestinal metaplasia of gastric mucosa are the most common conditions leading to the development of GC (15, 16), whereas serum pepsinogen (PG) and gastrin-17 (G-17) levels are usually used as biomarkers for the gastric mucosal atrophy status (17). The “serology biopsy” has proved to be useful in the screening for GC high-risk populations who experience gastric mucosal atrophy, which is defined as the combined detection of Hp antibody, PG, and G-17 (18).

The combination of non-invasive serological screening and endoscopy is helpful to improve the effect of GC screening (19). Therefore, the GC risk score scale (GCRSS) based on the serological indicators of Hp infection and atrophy was developed and recommended by the National Health Commission of China in 2022 as a screening strategy for GC in high-risk populations (20). Understanding the trade-offs in lifetime benefits, harm, and cost between current guidelines and alternative screening strategies is necessary to be integrated into dialogues on cancer control policy. The objective of our study was to assess the clinical benefits, harm, cost, and cost-effectiveness of the GCRSS screening strategy from a Chinese healthcare system perspective.

## Materials and methods

### Overview

A previously developed microsimulation model was adapted to evaluate the GCRSS strategy, in which 100,000 individuals were simulated from the age of 20 for each GC screening scenario (21). Because of the lack of data on the willingness-to-pay (WTP) of China, we assumed that the WTP threshold was equal to three times the per capita gross domestic product (GDP) in China, according to the World Health Organization cost-effectiveness definition (22). An incremental cost-effectiveness ratio (ICER) < one time the per capita GDP was defined as highly cost-effective; 1 to 3 times the per capita GDP was defined as cost-effective; and an ICER >three times the per capita GDP was defined as not cost-effective. In 2021, the per capita GDP in China was $12,552 (23).

The primary outcomes were GC incidence reduction, number of GC deaths averted, number of endoscopies, number of cause-specific deaths, costs, life years, and quality-adjusted life years (QALYs). We calculated the ICER by the difference in costs divided by the difference in QALYs. Moreover, the benefit-to-harm ratio was measured, which was defined as the ratio of screening complication-related events to GC deaths prevented.

The model was implemented using TreeAge Pro 2022 software (version 2022 R1.1; https://www.treeage.com), and additional statistical analyses were performed in R software (version 4.1.3; http://www.r-project.org).

### Modeling

**Figure 1:**
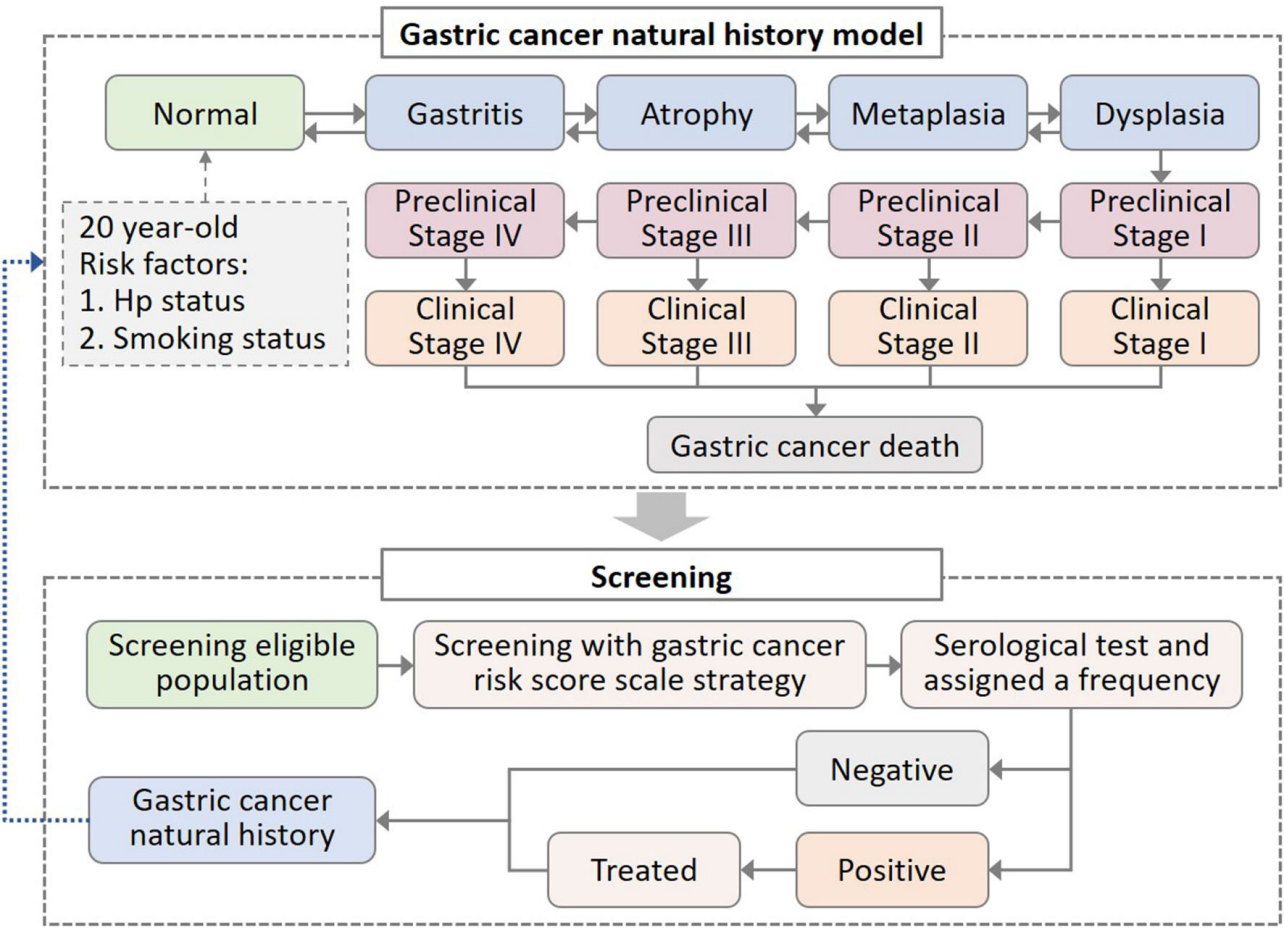
Decision-making model of gastric cancer (GC) natural history and screening intervention. In the natural history model of GC, all healthy states suffered background mortality, while clinical gastric cancer states suffered additional GC mortality. Hp, *Helicobacter pylori*.

We simulated the natural history of GC by constructing a model with 15 health states (Figure 1). The model adopted a 1-year cycle length and a lifetime horizon. Through several precancerous lesion states, individuals can progress to more advanced precancerous lesions and eventually invasive GC. In the absence of screening, we assumed that individuals were diagnosed only when developing clinical symptoms. The screening was performed to diagnose the precancerous and preclinical patients. After the intervention of screening strategies, the GC natural history would be altered due to the detection or treatment of precancerous lesions or preclinical cancer.

We considered the impact of individual risk profiles on disease progression, such as Hp infection and smoking behavior, which were the two most powerful risk factors for GC (10, 24–26). We assumed that Hp infection and smoking would increase the risk of progression to atrophy and dysplasia, respectively. Individuals entered the model from the age of 20 and were assigned Hp infection status and smoking behavior (Figure 1). Based on epidemiologic data, we applied 67% Hp prevalence and 64 and 34% smoking rate in men and women, respectively (27, 28). According to the smoking intensity, current smokers were further divided into <10 cigarettes per day and ≥10 cigarettes per day (29, 30).

Due to a paucity of progression rates data for the potential biological process from normal to GC, our model was calibrated to epidemiological data of precancerous lesions prevalence, GC incidence, and GC stage-specific proportion (31–33), to obtain these parameter sets of transition probabilities (more details were described in the Supplementary materials). We extracted background mortality that was specific for age and sex from Chinese life tables, and derived stage-specific GC mortality from a published follow-up study (34, 35). We validated the model using data not used in the calibration. Our model outcomes showed that the relative risk of GC incidence associated with Hp infection positive (4.3) and smoking (1.8) was matched with published estimates (95% *CI*, 2.7–7.2 and 1.5–1.8, respectively) (26, 36).

### Strategies

Compared with no screening, we evaluated seven strategies of the GCRSS with varying starting ages, such as 40, 45, 50, 55, 60, 65, and 70 years. For all the strategies, individuals were screened for the first time at the corresponding starting screening age. The target population for the GCRSS screening strategy was the GC high-risk population, who aged 40 years or older with one of the following conditions (residing in high-incidence areas, Hp infection, previous precancerous diseases, a positive family history of GC, regular intake of high-salt diet, smoking, and heavy alcohol drinking, etc.) (37).

The GCRSS strategy stratified individuals according to the serological detection results of serum PG, G-17, and Hp, followed by different scheduling endoscopy accordingly (37). The tested individuals were divided into four GC risk levels with stepwise increased GC risk: level A, Hp (–) and atrophy (–); level B, Hp (+) and atrophy (–); level C, Hp (+) and atrophy (+); and level D, Hp (–) and atrophy (+) (Supplementary Figure S3). Individuals with level A were retested for the Hp and atrophy status every 5 years, while level D would undergo annual endoscopic screening. Level B and C would receive triennial and biennial endoscopic screening, respectively, following standard quadruple therapy for Hp eradication (1,000 mg amoxicillin, 500 mg clarithromycin, 20 mg esomeprazole, 220 mg bismuth potassium citrate, twice daily for 2 weeks) (38).

According to the GC treatment guideline in China, individuals with an endoscopy result of dysplasia or asymptomatic GC stage I were subsequently offered an endoscopic submucosal dissection (ESD) or surgery to remove the mucosal lesions (9). The screening-related complications of bleeding and perforation were considered in ESD and surgery, and we assumed that individuals undergoing surgery would suffer surgery-related death. The corresponding progression risk was assumed to be decreased after ESD or surgery (39). We considered the characteristics of screening methods, such as sensitivity and specificity (40, 41). In the model, we assumed perfect adherence in all scenarios. The baseline values and plausible ranges of model parameters were listed in Supplementary Table S1.

### Cost and utility estimates

We considered direct medical costs, such as serological tests costs; treatment costs for Hp, complications, and GC; endoscopic costs; and surgical procedures costs, which were obtained from local hospital (pricing of a local hospital, which was set by local governments according to national regulations) and published studies (Supplementary Table S1). All costs are reported in 2021 US dollars (1 USD = 6.4856 RMB, in 2021) (42).

Quality-adjusted life years were used to evaluate the health outcomes of each strategy and were defined as the survival time adjusted by the health utility. We assumed a utility score of 1 for normal and precancerous states because there is no available data on the quality of life of patients with GC precancerous lesions (43). The utility values for the four GC stages were derived from a Chinese population-based study (Supplementary Table S1) (43). We discounted costs and QALYs at 5% per year according to the China Guidelines for Pharmacoeconomic Evaluations (44).

### Sensitivity analysis

To explore the effects of parameter uncertainty, we conducted univariate sensitivity analyses for all parameters, and the plausible range was either determined by ±20% of the base value or based on the reported 95% uncertainty bounds (Supplementary Table S1). Additionally, probabilistic sensitivity analysis was performed based on 1,000 Monte Carlo simulations to estimate the combined uncertainty of all parameters and the probability of the strategy being cost-effective. To further evaluate the model robustness, we also conducted additional subgroup and scenario analyses.

## Results

### Clinical outcomes

**Figure 2:**
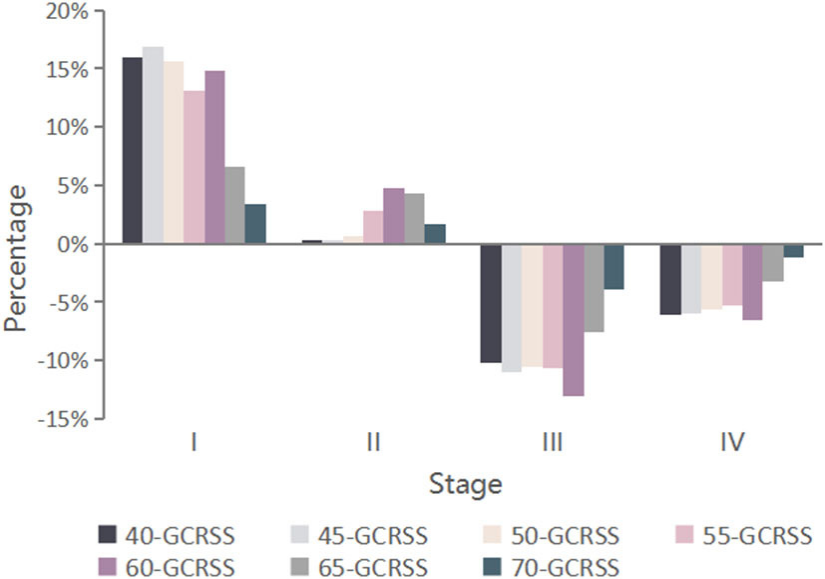
Changes in GC stage distribution for seven alternative screening strategies compared with no screening. GCRSS, gastric cancer risk score scale.

Among the 100,000 hypothetical individuals aged 20 years followed up over a lifetime, there were 9,995 GC-related deaths in the unscreened cohort, with 11.6% GC incidence. For the 7 GCRSS strategies, as the starting screening age increased, the relative reduction in GC incidence and the estimated number of GC deaths prevented decreased from 70.6 to 15.4% and 7,374 to 1,416, respectively (Table 1 and Supplementary Figure S4). Furthermore, compared with no screening, the GC stage distribution of all strategies was significantly improved, with a substantially decrease in advanced GC stages (Figure 2). The proportion of GC stages III and IV increased slightly with increasing screening start age (Supplementary Figure S8).

**Table 1:** Clinical and cost-effectiveness outcomes.

**Strategy**	**No screening**	**40-GCRSS**	**45-GCRSS**	**50-GCRSS**	**55-GCRSS**	**60-GCRSS**	**65-GCRSS**	**70-GCRSS**
GC incidence reduction^a^, %	NA	70.6	67.2	62.1	56.0	33.0	21.9	15.4
GC deaths averted^b^	NA	7,374	6,997	6,495	5,848	3,639	2,208	1,416
Life-years^c^	56.060	56.855	56.827	56.707	56.625	56.336	56.152	56.110
Number of endoscopies^b^	NA	1,01,8390	8,81,227	7,29,442	5,87,214	4,55,496	3,25,224	2,14,120
Life-years gained per GC deaths averted	NA	10.8	11.0	10.0	9.7	7.6	4.2	3.5
Endoscopy screenings per GC deaths averted	NA	138	126	112	100	125	147	151
Endoscopy screenings per life-years gained	NA	13	11	11	10	16	35	43
NNS to prevent 1 GC death	NA	14	14	15	17	27	45	71
Complication-related deaths	NA	166	163	187	150	149	129	93
Net number of deaths averted ^d^	NA	7,208	6,834	6,308	5,698	3,490	2,079	1,323
Benefit-to-harm ratio	NA	0.392	0.416	0.430	0.464	0.651	0.909	1.181
Costs^c^^,^ ^e^, $	103.6	960.3	834.1	700.0	566.6	459.0	349.8	267.9
QALYs^c^^,^ ^e^	19.293	19.361	19.357	19.345	19.338	19.314	19.302	19.300
**ICER ($/QALY)**								
Vs. no screening	NA	12,601	11,476	11,464	10,315	17,164	27,446	22,420
Vs. previous^f^	NA	29,115	14,254	ED	10,315	ED	ED	ED

a Compared with no screening.

b Per 1,00,000 individuals.

c Per-person averages.

d Number of GC deaths prevented minus number of complication-related deaths.

e Discounted at an annual rate of 5%.

f Compared with previous less costly strategy.

GCRSS, gastric cancer risk score scale; GC, gastric cancer; NNS, the number needed to screen; QALYs, quality-adjusted life years; ICER, incremental cost-effectiveness ratio; NA, not applicable; ED, extended dominated (less effective and less cost-effective than a more expensive strategy).

Screening with the GCRSS strategy at the age of 40 years (40-GCRSS), the lifetime number of endoscopies was 1,018,390 per 100,000 individuals (Table 1). Although the 40-GCRSS strategy resulted in the greatest number of endoscopies, it provided the largest life-year gain and the greatest number of GC deaths averted among all strategies. We also calculated the incremental endoscopy screenings and life-years gained ratio by incremental endoscopy screenings divided by incremental life-years gained. The 40-GCRSS strategy was associated with the largest ratio of 49 endoscopy screenings per life-years gained (Supplementary Figure S6).

As another way to examine the strategies, the benefit-to-harm ratio was calculated. Screening from age 40 years would have the lowest benefit-to-harm ratio of 0.392. In contrast, there were more complication-related cases than GC deaths averted when screening was started at age 65 or older (Table 1).

### Cost-effectiveness analysis

Compared with no screening, GCRSS screenings were associated with additional QALYs ranging from 0.007 to 0.068 at additional costs ranging from $164.3 to $856.7, giving ICERs ranging from $10,315 to $27,446 per QALY gained (Table 1). All the ICERs were lower than three times per capita GDP ($37,655 per QALY).

**Figure 3:**
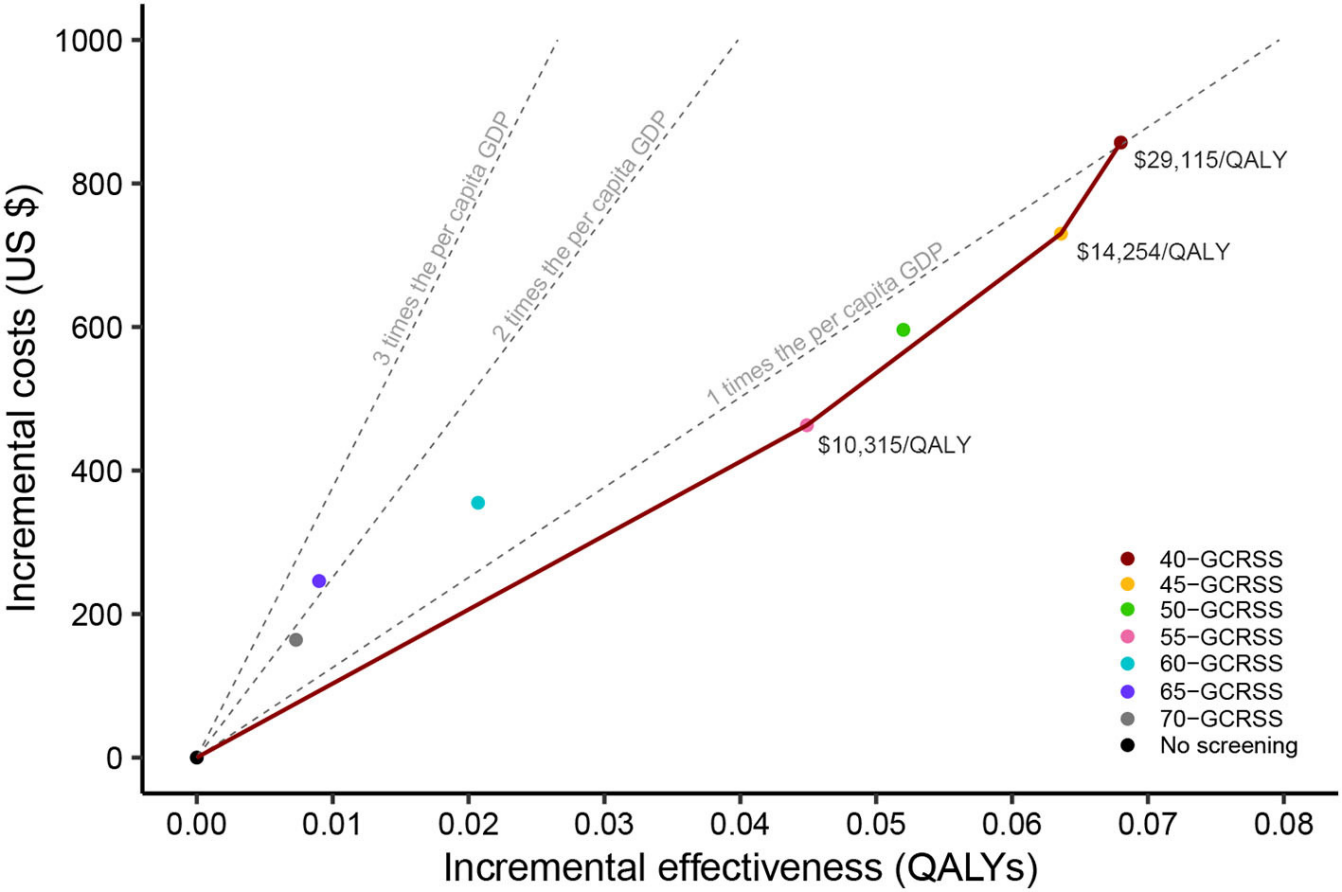
Cost-effectiveness frontier of seven alternative screening strategies and no screening. QALYs, quality-adjusted life years; GDP, gross domestic product; GCRSS, gastric cancer risk score scale.

Further comparisons across all strategies were performed, and we calculated the corresponding cost-effectiveness frontier curves (Figure 3). This frontier was comprised of four strategies: the no screening strategy, the 55-GCRSS strategy, the 45-GCRSS strategy, and the 40-GCRSS strategy. At a WTP threshold of three times per capita GDP ($37,655 per QALY), although the 40-, 45-, and 55-GCRSS strategies were all cost-effective, the 40-GCRSS strategy was considered to be the leading cost-effective strategy because of its better effect.

### Sensitivity analysis

**Figure 4:**
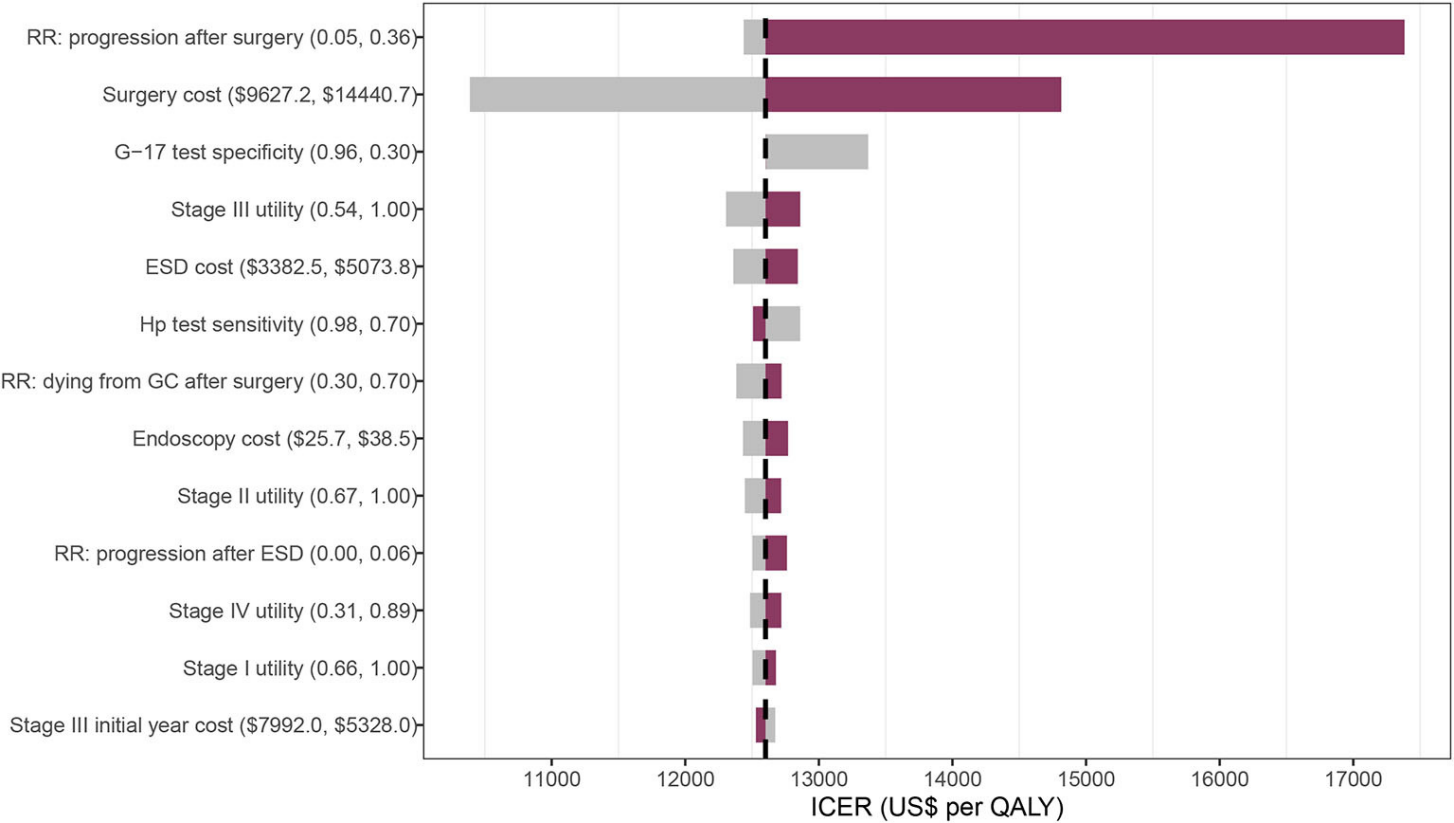
Tornado diagram on one-way sensitivity analysis of the 40-GCRSS strategy compared with no screening. RR, relative risk; G-17, gastrin-17; ESD, endoscopic submucosal dissection; Hp, *Helicobacter pylori*; ICER, incremental cost-effectiveness ratio; QALY, quality-adjusted life year.

Univariate sensitivity analyses found that the results were largely unchanged under the changes of each parameter. The relative risk of progressing from dysplasia after surgery and the surgery cost generated a significant influence on the ICER compared with no screening (Figure 4).

**Figure 5:**
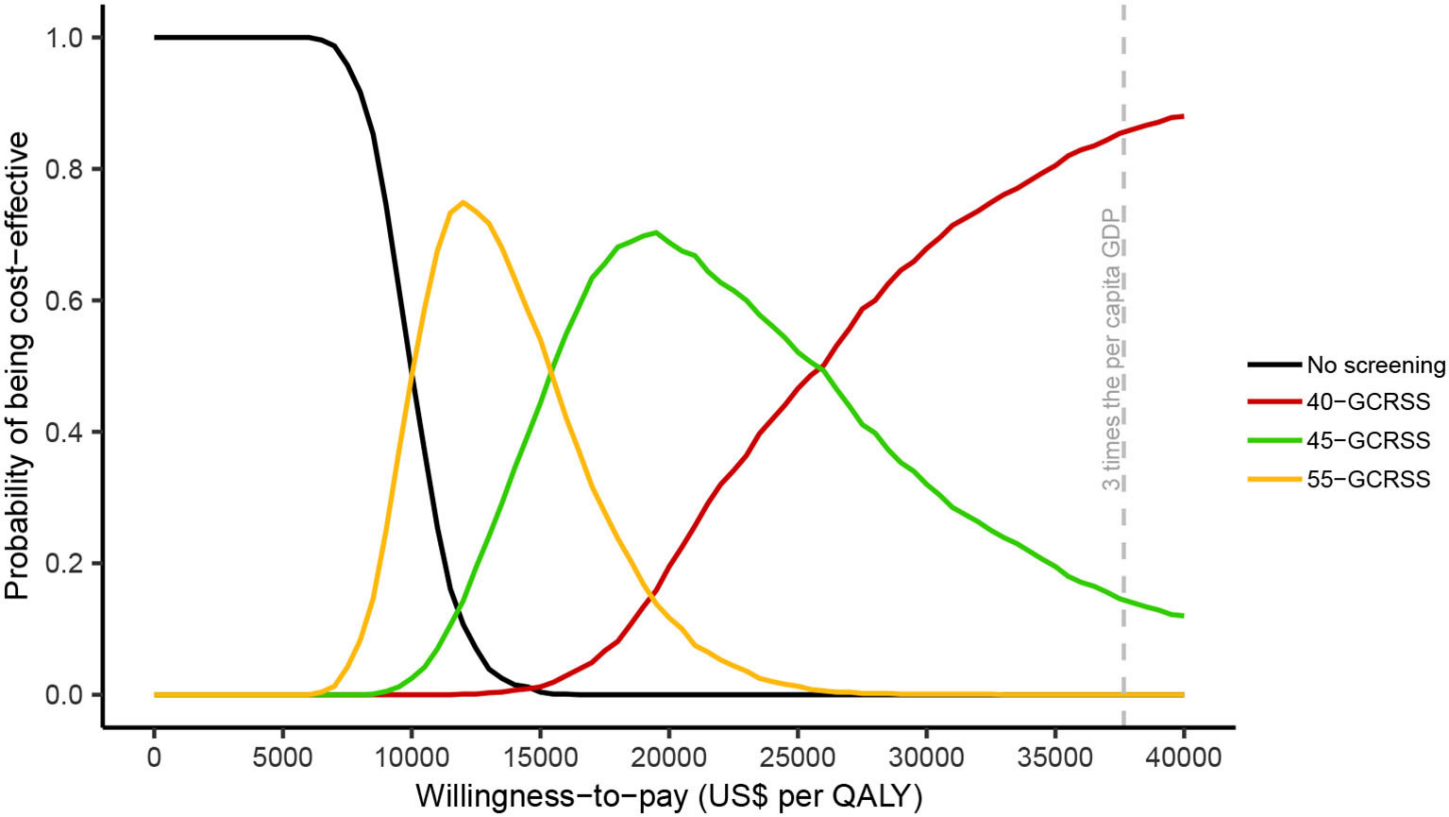
Cost-effectiveness acceptability curves. QALY, quality-adjusted life year; GDP, gross domestic product; GCRSS, gastric cancer risk score scale.

In the probabilistic sensitivity analyses, for a WTP between $0 and $6,300 per QALY, no screening was the most cost-effective strategy (Figure 5). When WTP was increased between $9,990 and $15,390 per QALY, screening with GCRSS from age 55 had the highest probability of being cost-effective. The 45-GCRSS strategy was the most cost-effective screening strategy in the WTP threshold range from $15,480 to $25,920 per QALY. Additionally, at a threshold of $26,010 and $37,655 per QALY, the 40-GCRSS strategy outperformed other strategies and showed 0.502 and 0.856 probability of being cost-effective, respectively.

In the three smoking subgroups, the relative reduction in GC mortality was similar between the 40- and 45-GCRSS strategy, which was slightly lower than that in the Hp+ subgroups (Supplementary Figure S4). Obviously, almost all clinical outcomes performed best in the current smokers and Hp+ subgroups (Supplementary Table S3). Compared with no screening, the GC stage distribution changed significantly in stages I, III, and IV in subgroups (Supplementary Figure S7). In addition, screening in current smokers was more cost-effective than in the overall population or other subgroups (Supplementary Table S3).

## Discussion

We constructed a well-calibrated model to simulate the GC progression over the lifetime course under seven screening scenarios, and estimated the corresponding clinical benefits, harms, and cost-effectiveness over the lifetime course. The base case results of our model-based study suggested that screening with the GCRSS strategy from age 40 could improve the clinical benefits, benefit-to-harm ratio, and the cost-effectiveness of GC screening among all the strategies, with an ICER of $29,115 per QALY compared with the 45-GCRSS strategy below the WTP threshold of $37,655 per QALY.

To our knowledge, this is the first study based on a calibrated microsimulation model to comprehensively evaluate the long-term clinical and economic consequences of GC screening strategies. The previous studies mainly evaluated the cost-effectiveness of endoscopic screening programs directly for GC high-risk populations (45, 46). Actually, most of the high GC risk areas in China are rural, with scarce medical resources or limited endoscopic screening facilities (47, 48). If all individuals at high risk of GC undergo endoscopic screening, the endoscopy resources will not be enough to meet the huge demand (49). In addition, endoscopic screening is an invasive examination with poor repeatability and low compliance, which is not conducive to the early detection of GC, so that it is limited in the popularization of screening. Serological diagnosis of Hp and atrophy, as preferable to non-invasive measurement, can provide a more acceptable way for the detection of early GC. Although the serological test prior to endoscopy may entail additional cost, this cost may be offset by the reduction of GC disease burden caused by less endoscopy and higher compliance.

Different strategies have benefits and harms, thus the optimal strategy may depend on the acceptable trade-off. We found that the earlier the age of starting screening, the more screening-related adverse consequences are caused, but the more corresponding benefits are also obtained. The 40-GCRSS strategy was associated with a lower benefit-to-harm ratio of 0.392, while the ratio would exceed 1 as the starting age increased to 65. This means that there were more cases of screening-related adverse events than GC deaths prevented when screening was targeted to individuals aged 65 or older. Considering the balance of benefits and harms, it is not appropriate to start implementing the GCRSS strategy after the age of 65. China has a vast territory, and the level of economic development in different regions is uneven (50). Likewise, when choosing appropriate screening strategies, decision-makers should consider the local economic level and GC disease burden. Compared with the 40-GCRSS strategy, the 45-GCRSS strategy had a similar GC incidence improvement and the highest probability of being cost-effective at a WTP ranging from $15,480 to $25,920 per QALY. Consequently, if appropriate, it is preferable to set the starting screening age at 45 years for areas with limited health resources and underdeveloped economies.

With the implementation of the Healthy China 2030 initiative, the Hp infection rate and smoking rate in China will reach the goal of <20% (51, 52). Our scenario analysis results demonstrated that the ICERs of the 40-GCRSS strategy remained lower than $37,655 per QALY WTP threshold compared with no screening, despite the Hp infection rate and smoking rate decreased (Supplementary Table S4). The simultaneous implementation of actions to reduce GC risk factors and cost-effective GC screening strategies will facilitate the reduction of the GC disease burden in China.

Eradication of Hp infection can improve the gastric mucosal inflammatory response, and prevent or delay the progression of atrophy or intestinal metaplasia (53). Hp infection is considered to be the most important and controllable risk factor for the prevention of GC, thus eradicating Hp should be the primary preventive measure (14, 54). Our study found that compared with Hp– subgroup, the GCRSS strategy screening for Hp+ individuals would provide Hp eradication therapy, which significantly improves the GC distribution and reduces the incidence and mortality of GC. The GCRSS strategy combined Hp detection and eradication with endoscopic screening, which obtained additional benefits from eradicating risk factors than endoscopic screening alone. Furthermore, it also provided individuals with appropriate endoscopy screening frequency according to the Hp infection status, which improved their compliance with endoscopy. We changed the effect of the progression relative risk for surgery for a further sensitivity analysis. When the relative risk was lower than 0.9, the ICER of the 40-GCRSS strategy was lower than the $37,655 per QALY threshold compared with no screening (Supplementary Figure S9). Even if there is little to no improvement after surgery, it remains a high-value strategy for improving cancer outcomes. The traditional method for the treatment of early GC is surgical resection, after which the 5-year survival rate can reach more than 90% (55). However, surgery destroys the normal anatomical structure of the stomach and affects the long-term physiological function of the patient. ESD is superior to surgery in safety and effectiveness (56), and it is recommended by the guidelines as the preferred alternative for dysplasia or early GC (57). If ESD treatment is mature and widespread, the corresponding screening strategy will be more effective and cost-effective.

This study has limitations. First, utility values of precancerous lesions states might not accurately represent the patients' quality of life. Patients with precancerous lesions may have a worse quality of life than the general population, while the reasons for the decrement are unclear and the evidence is limited (58). Second, we used perfect adherence to all scenarios. However, this assumption provided the model with the ability to predict the maximum achievable benefits of public health strategies. Third, although there are many factors that may play a role in the development of GC, smoking and Hp factors, which we considered in our model, are the two strongest risk factors for GC (25, 26). Finally, future studies can further compare the GCRSS strategy with different GC screening strategies to explore the more suitable GC screening strategies for the China setting.

## Conclusions

This modeling study suggested that from the Chinese healthcare system perspective, the GCRSS strategy screening from age 40 was an effective and leading cost-effective strategy in China. The findings provide an important basis for policymakers to formulate and optimize GC prevention and control policies in China.

## Data availability statement

The original contributions presented in the study are included in the article/Supplementary material, further inquiries can be directed to the corresponding author/s.

## Author contributions

SQ, XWang, XWan, CT, and XZ conceived and designed the study. SQ, XWan, XWang, SL, MW, YP, and LW developed the economic model, collected the data, performed the analyses, and interpreted the results. CT, XZ, and XWang supervised the analyses. SQ and XWan drafted and critically revised the manuscript for important intellectual content and approved the version to be published. All authors had full access to all the data and take responsibility for the integrity of the data and reviewed and approved the final version.

## Funding

This work was supported by a grant from the National Natural Science Foundation of China (grant numbers 71874209 and 82073818) and the research project of the Health Commission of Hunan province (grant number 202113050283). The funders had no role in study design, data collection and analysis, decision to publish, or preparation of the manuscript.

## Conflict of interest

The authors declare that the research was conducted in the absence of any commercial or financial relationships that could be construed as a potential conflict of interest.

## Publisher's note

All claims expressed in this article are solely those of the authors and do not necessarily represent those of their affiliated organizations, or those of the publisher, the editors and the reviewers. Any product that may be evaluated in this article, or claim that may be made by its manufacturer, is not guaranteed or endorsed by the publisher.
