# Supplementary material for: Benefit-to-harm ratio and cost-effectiveness of government-recommended gastric cancer screening in China: A modeling study
Source: Front Public Health. 2022 Aug 10;10:955120. doi: 10.3389/fpubh.2022.955120 (PMC9399635; doi:10.3389/fpubh.2022.955120)
Supplement: Supplementary file 1 [file Data_Sheet_1.docx]

**Supplementary appendix**

**Benefit-to-harm ratio and cost-effectiveness of government-recommended gastric cancer screening in China: a modeling study**

Shuxia Qin, MD; Xuehong Wang, PhD; Sini Li, MD; Chongqing Tan, PhD; Xiaohui Zeng, PhD; Meiyu Wu, MD; Ye Peng, BS; Liting Wang, MD; Xiaomin Wan, PhD

**Contents**

**Table S1.** Model inputs

**Table S2.** Natural history parameters for calibration

**Table S3.** Clinical and cost-effectiveness outcomes in subgroups

**Table S4.** Scenario analyses of Hp infection rate and smoking rate

**Figure S1.** Results of model calibration

**Figure S2.** Model predicted and observed stage distribution of GC

**Figure S3.** Flowchart of gastric cancer risk score scale strategy

**Figure S4.** Relative reduction in GC incidence and mortality compared with no screening in overall cohort and subgroups

**Figure S5.** Proportion of screening-detected GC in overall cohort

**Figure S6.** Incremental endoscopy screenings and incremental life-years gained in overall cohort for all strategies

**Figure S7.** Changes in GC stage distribution in subgroups compared with no screening

**Figure S8.** GC stage distribution of GCRSS strategies with different starting screening ages in overall cohort

**Figure S9.** Sensitivity analysis of the relative risk of progressing after surgery for 40-GCRSS strategy compared with no screening

| **Table S1. Model inputs** | | | | |
| --- | --- | --- | --- | --- |
| Parameter | Base case | Range | Distribution | Reference |
| Screening sensitivity |  |  |  |  |
| Hp | 0.85 | 0.70-0.98 | Beta | ^[1, 2]^ |
| PG | 0.71 | 0.59-0.82 | Beta | ^[3, 4]^ |
| G-17 | 0.86 | 0.76-0.97 | Beta | ^[5]^ |
| Endoscopy | 0.81 | 0.78-0.84 | Beta | ^[6, 7]^ |
| Screening specificity |  |  |  |  |
| Hp | 0.79 | 0.63-0.94 | Beta | ^[1, 2]^ |
| PG | 0.98 | 0.97-0.99 | Beta | ^[3, 4]^ |
| G-17 | 0.93 | 0.30-0.96 | Beta | ^[5]^ |
| Endoscopy | 1.00 | 0.99-1.00 | Beta | ^[6, 7]^ |
| Outcomes |  |  |  |  |
| Relative risk of ESD |  |  |  | ^[8]^ |
| Progression from dysplasia | 0.02 | 0.00-0.06 | Fixed |  |
| Dying from GC | 0.00 | 0.00-0.06 | Fixed |  |
| Relative risk of surgery |  |  |  | ^[8]^ |
| Progression from dysplasia | 0.06 | 0.05-0.36 | Fixed |  |
| Dying from GC | 0.53 | 0.30-0.70 | Fixed |  |
| Complication of ESD | 0.0321 | Fixed | Beta | ^[9, 10]^ |
| Complication of surgery | 0.0918 | Fixed | Beta | ^[9]^ |
| Dying from surgery | 0.0069 | Fixed | Beta | ^[11]^ |
| Costs, $USD |  |  |  |  |
| Screening cost |  |  |  | local charge |
| Hp | 6.94 | 5.55-8.33 | Gamma |  |
| PG | 15.42 | 12.34-18.50 | Gamma |  |
| G-17 | 15.42 | 12.34-18.50 | Gamma |  |
| Endoscopy | 32.10 | 25.68-38.52 | Gamma |  |
| Treatment cost |  |  |  |  |
| Hp eradication | 109.29 | 87.43-131.15 | Gamma | local charge |
| ESD | 4228.14 | 3382.51-5073.77 | Gamma | ^[12]^ |
| Surgery | 12033.94 | 9627.15-14440.73 | Gamma | ^[12]^ |
| Complication | 151.46 | 121.17-181.75 | Gamma | ^[13]^ |
| GC initial year |  |  |  | ^[14]^ |
| Stage I | 9249.37 | 7399.50-11099.24 | Gamma |  |
| Stage II | 5791.64 | 4633.31-6949.97 | Gamma |  |
| Stage III | 6660.00 | 5328.00-7992.00 | Gamma |  |
| Stage IV | 4328.32 | 3462.66-5193.98 | Gamma |  |
| Annual health care |  |  |  | ^[14]^ |
| Stage I and II | 409.00 | 327.20-490.80 | Gamma |  |
| Stage III and IV | 435.00 | 348.00-522.00 | Gamma |  |
| Utility weight |  |  |  | ^[15]^ |
| Stage I | 0.85 | 0.66-1.00 | Beta |  |
| Stage II | 0.86 | 0.67-1.00 | Beta |  |
| Stage III | 0.79 | 0.54-1.00 | Beta |  |
| Stage IV | 0.60 | 0.31-0.89 | Beta |  |
| ESD with complication | -1 week | Fixed | Fixed | ^[16]^ |
| Surgery with complication | -1 month | Fixed | Fixed | ^[16]^ |
| local charge: pricing of a local hospital, which were set by local governments according to national regulations.  Hp, Helicobacter pylori; PG, serum pepsinogen; G-17, gastrin-17; ESD, endoscopic submucosal dissection; GC, gastric cancer. | | | | |

**Model calibration**

We established initial plausible ranges for all model input parameters, and then used a differential evolution algorithm to search the parameter sets in the plausible ranges^[17]^. A total of 38 calibration targets were used, including 24 age-specific prevalence of gastritis, atrophy, metaplasia, and dysplasia, 10 age-specific GC incidence and 4 GC stage-specific proportion.^[14, 18, 19]^ We applied the likelihood approach to identify a series of good fitting parameter sets^[17]^. The model outputs showed good agreement to the calibration targets, and the calibration results were showed in supplementary Table S2, Figure S1, and Figure S2.

| **Table S2.** **Natural history parameters for calibration** | | |
| --- | --- | --- |
| Parameter^a^ | Plausible range | Reference |
| Transition probabilities |  |  |
| Progression |  |  |
| Normal to gastritis | 0.021571-0.064288 | ^[20-22]^ |
| Gastritis to atrophy | 0.002821-0.018955 | ^[23-28]^ |
| Atrophy to metaplasia | 0.284554-0.492972 | ^[20, 22, 29, 30]^ |
| Metaplasia to dysplasia | 0.025706-0.041946 | ^[25, 27, 31, 32]^ |
| Dysplasia to preclinical stage I |  | ^[31-35]^ |
| Age 20-24 | 0.000001-0.000094 |  |
| Age 25-29 | 0.000002-0.000093 |  |
| Age 30-34 | 0.002403-0.009166 |  |
| Age 35-39 | 0.0001370058-0.005453 |  |
| Age 40-44 | 0.004981-0.009413 |  |
| Age 45-49 | 0.00254-0.008945 |  |
| Age 50-54 | 0.004285-0.010886 |  |
| Age 55-59 | 0.030797-0.064041 |  |
| Age 60-64 | 0.010762-0.031133 |  |
| Age 65-69 | 0.01205-0.020047 |  |
| Age 70-74 | 0.014522-0.060257 |  |
| Age 75-79 | 0.020595-0.098928 |  |
| Age 80-89 | 0.01425-0.09999711 |  |
| Preclinical stage I to preclinical stage II | 0.119749-0.341397 | ^[36-38]^ |
| Preclinical stage II to preclinical stage III | 0.142197-0.446995 | ^[37, 39]^ |
| Preclinical stage III to preclinical stage IV | 0.101054-0.667976 | ^[39]^ |
| Preclinical stage I to clinical stage I | 0.031403-0.111953 | ^[40]^ |
| Preclinical stage II to clinical stage II | 0.050249-0.176599 | ^[40]^ |
| Preclinical stage III to clinical stage III | 0.169992-0.79205 | ^[40]^ |
| Preclinical stage IV to clinical stage IV | 0.101914-0.76749 | ^[40]^ |
| Regression |  |  |
| Gastritis to normal | 0.005258-0.025584 | ^[20, 21, 26]^ |
| Atrophy to gastritis | 0.00331-0.161254 | ^[20, 27, 29, 41]^ |
| Metaplasia to atrophy | 0.001518-0.020927 | ^[26, 28, 29, 32]^ |
| Dysplasia to metaplasia | 0.01093735-0.051122 | ^[28, 31, 41]^ |
| Risk factors on disease progression, relative risk | |  |
| Hp |  |  |
| Gastritis to atrophy | 3.123618-9.54744 | ^[42]^ |
| Smoking |  |  |
| Atrophy to metaplasia |  | ^[43]^ |
| Current smokers <10 cigarettes per day | 1.0064-1.293994 |  |
| Current smokers ≥10 cigarettes per day | 1.100261-1.69964 |  |
| Former smokers | 1.024005-1.493069 |  |
| Metaplasia to dysplasia |  | ^[43]^ |
| Current smokers <10 cigarettes per day | 1.01242-2.758659 |  |
| Current smokers ≥10 cigarettes per day | 2.229484-5.946374 |  |
| Former smokers | 1.004285-3.08775 |  |
| Hp, Helicobacter pylori.  ^a^Yearly transition probabilities. | | |

**Figure S1. Results of model calibration**

| A, gastritis prevalence | B, atrophy prevalence |
| --- | --- |
| 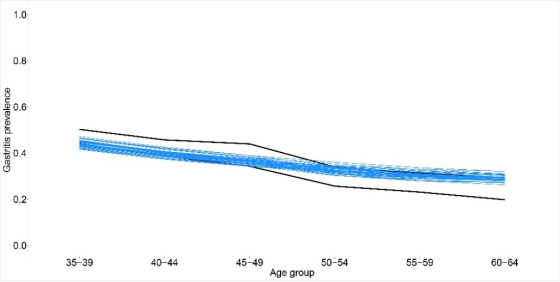 | 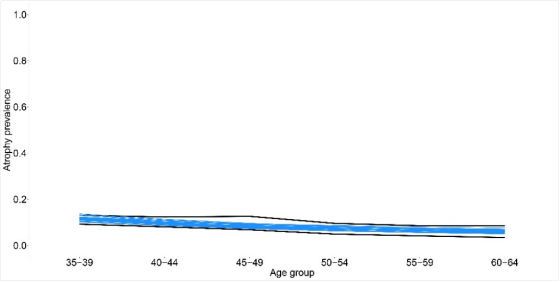 |
| C, metaplasia prevalence | D, dysplasia prevalence |
| 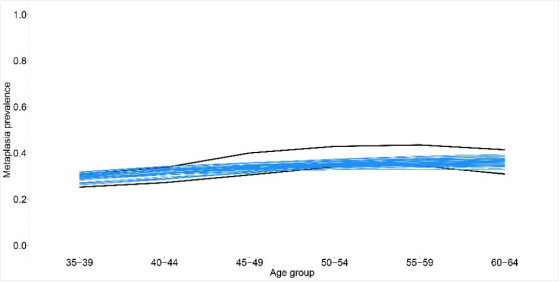 | 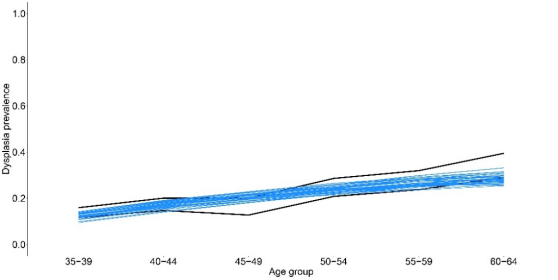 |
|  |  |
| E, GC incidence |  |
| 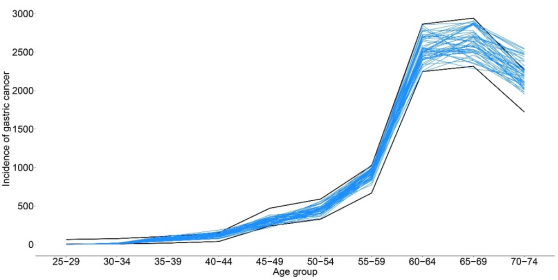 | |

Figure A, B, C and D, model output for gastritis, atrophy, metaplasia and dysplasia age-specific prevalence, respectively. Figure E, model output for GC age-specific incidence.

Black lines, 95% confidence intervals of prevalence or incidence; blue lines, model output for the top 50 best-fitted parameter sets. GC, gastric cancer.

**Figure S2. Model predicted and observed stage distribution of GC**


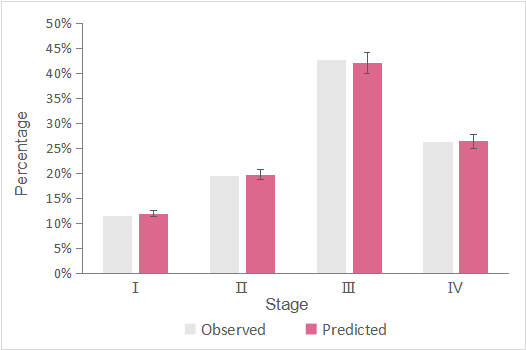


Blue bars, the observed stage distributions of GC; red bars, model prediction on stage distributions of GC; error bars, uncertainty intervals. GC, gastric cancer.

**Figure S3. Flowchart of gastric cancer risk score scale strategy**


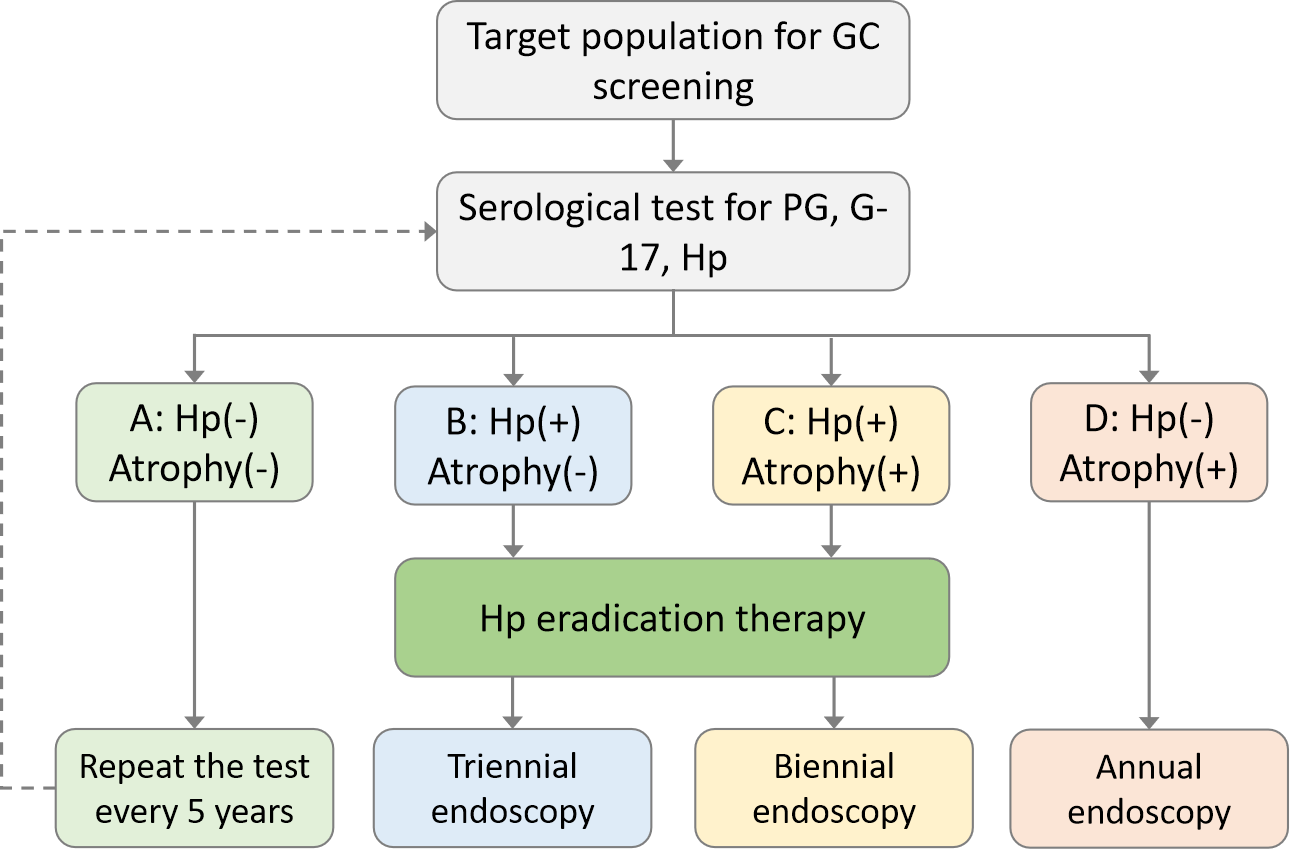


GC, gastric cancer; PG, serum pepsinogen; G-17, gastrin-17; Hp, Helicobacter pylori. -, negative; +, positive.

**Figure S4. Relative reduction in GC** **incidence and mortality compared with no screening in overall cohort and subgroups**

A, Relative reduction in GC incidence and mortality in overall cohort


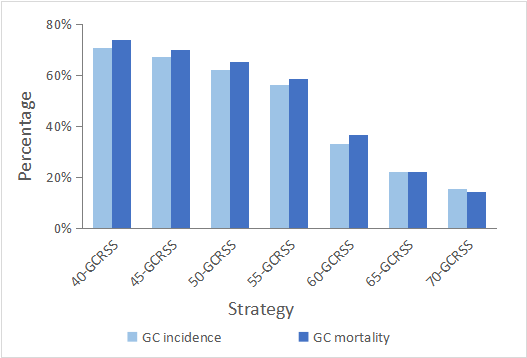


B, Relative reduction in GC mortality for 40- and 45-GCRSS strategy in subgroups


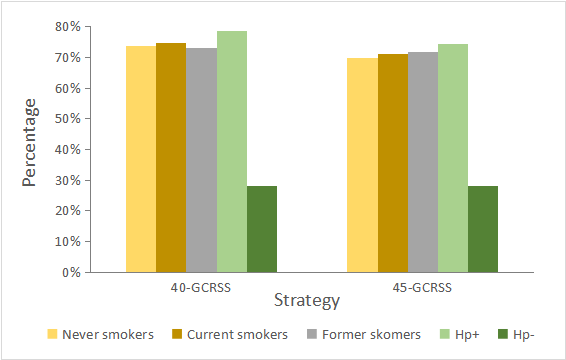


GCRSS, gastric cancer risk score scale; GC, gastric cancer; Hp, Helicobacter pylori.

**Figure S5.** **Proportion of screening-detected GC in overall cohort**


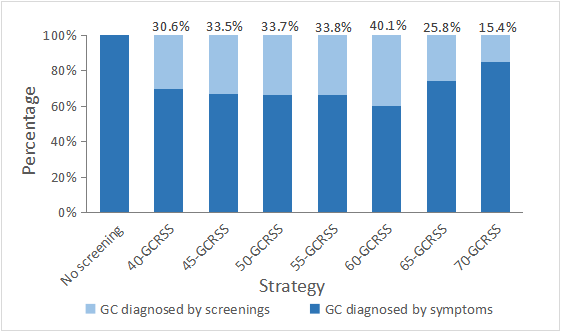


As the age of screening increases from 40 to 70, the proportion of screening-detected GC first increased and then decreased. The maximum proportion of screening-detected GC was associated with the 60-GCRSS strategy.

GCRSS, gastric cancer risk score scale; GC, gastric cancer.

**Figure S6. Incremental endoscopy screenings and incremental life-years gained in overall cohort for all strategies**


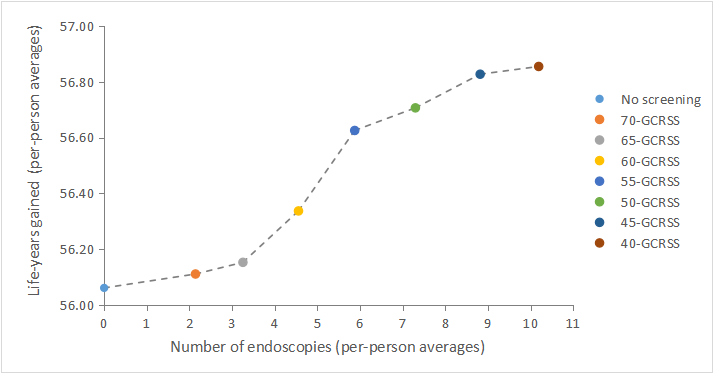


The larger the slope of the connecting line between the two points in the figure, the smaller the number of endoscopes required per life-years gained.

GCRSS, gastric cancer risk score scale.

**Figure S7. Changes in GC stage distribution in subgroups compared with no screening**


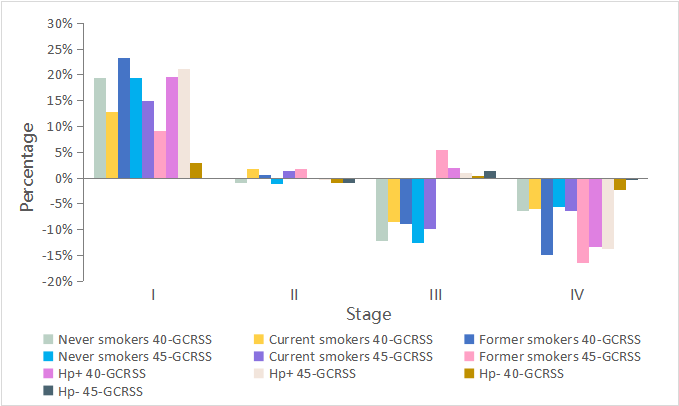


GCRSS, gastric cancer risk score scale; GC, gastric cancer; Hp, Helicobacter pylori.

**Figure S8. GC stage distribution of GCRSS strategies with different starting screening ages** **in overall cohort**


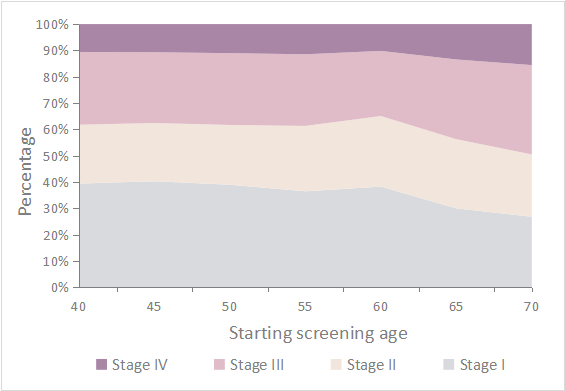


GCRSS, gastric cancer risk score scale.

| **Table S3. Clinical and cost-effectiveness outcomes in subgroups** | | | | | | | | | | |
| --- | --- | --- | --- | --- | --- | --- | --- | --- | --- | --- |
| Strategy in subgroup | Never smokers | | Current smokers | | Former smokers | | Hp+ status | | Hp- status | |
|  | 40-GCRSS | 45-GCRSS | 40-GCRSS | 45-GCRSS | 40-GCRSS | 45-GCRSS | 40-GCRSS | 45-GCRSS | 40-GCRSS | 45-GCRSS |
| GC incidence reduction^a^, % | 70.3 | 66.5 | 71.9 | 68.7 | 66.4 | 62.8 | 75.7 | 71.6 | 27.0 | 28.5 |
| Life-years^b^ | 58.443 | 58.419 | 55.856 | 55.823 | 56.171 | 56.062 | 56.774 | 56.699 | 57.022 | 57.089 |
| Life-years gained per GC deaths averted | 9 | 10 | 11 | 12 | 12 | 10 | 11 | 11 | 4 | 12 |
| Endoscopy screenings per GC deaths averted | 183 | 168 | 90 | 81 | 123 | 108 | 113 | 103 | 798 | 689 |
| Endoscopy screenings per life-years gained | 19 | 18 | 8 | 7 | 11 | 10 | 10 | 9 | 222 | 59 |
| NNS to prevent 1 GC death | 17 | 18 | 9 | 10 | 12 | 12 | 9 | 10 | 120 | 121 |
| Benefit-to-harm ratio | 0.447 | 0.475 | 0.334 | 0.355 | 0.321 | 0.231 | 0.373 | 0.403 | 0.886 | 0.728 |
| Costs^b,c^, $ | 786.6 | 687.0 | 1349.1 | 1164.4 | 1025.6 | 829.2 | 1319.3 | 1147.5 | 225.6 | 192.6 |
| QALYs^b,c^ | 19.603 | 19.600 | 19.381 | 19.374 | 19.407 | 19.374 | 19.350 | 19.341 | 19.383 | 19.389 |
| ICER ($/QALY)^d^ | 1771 | 16395 | 11281 | 10233 | 9884 | 12156 | 12170 | 11540 | 22225 | 10737 |
| ^a^Compared with no screening.  ^b^Per-person averages.  ^c^Discounted at an annual rate of 5%.  ^d^Vs. no screening  GCRSS, gastric cancer risk score scale; Hp, Helicobacter pylori; GC, gastric cancer; NNS, the number needed to screen; QALYs, quality-adjusted life years; ICER, incremental cost-effectiveness ratio; ICER, incremental cost-effectiveness ratio. | | | | | | | | | | |

| **Table S4. Scenario analyses of Hp infection rate and smoking rate** | |
| --- | --- |
| Scenarios | ICER ($/QALY, 40-GCRSS vs. no screening) |
| 20% Hp infection rate | 12536 |
| 20% smoking rate | 13564 |
| 20% Hp infection and smoking rate | 13521 |
| Hp, Helicobacter pylori; ICER, incremental cost-effectiveness ratio; QALY, quality-adjusted life year; GCRSS, gastric cancer risk score scale. | |

**Figure S9. Sensitivity analysis of the relative risk of progressing after surgery for 40-GCRSS strategy compared with no screening**


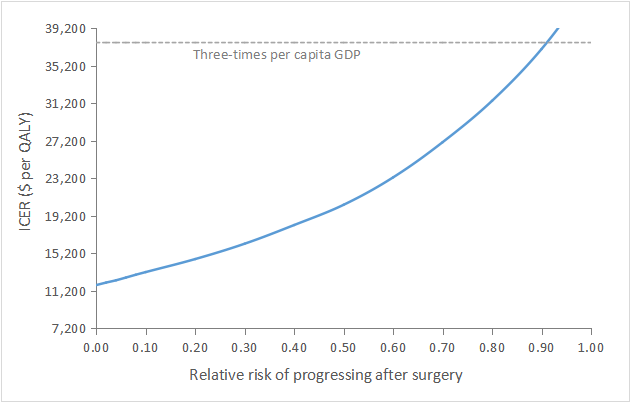


ICER, incremental cost-effectiveness ratio; QALY, quality-adjusted life year; GDP, gross domestic product.

**References**

1. Burucoa C, Delchier JC, Courillon-Mallet A, et al. Comparative evaluation of 29 commercial Helicobacter pylori serological kits[J]*.* Helicobacter, 2013; 18(3): 169-179.

2. Loy CT, Irwig LM, Katelaris PH, et al. Do commercial serological kits for Helicobacter pylori infection differ in accuracy? A meta-analysis[J]*.* Am J Gastroenterol, 1996; 91(6): 1138-1144.

3. Storskrubb T, Aro P, Ronkainen J, et al. Serum biomarkers provide an accurate method for diagnosis of atrophic gastritis in a general population: The Kalixanda study[J]*.* Scand J Gastroenterol, 2008; 43(12): 1448-1455.

4. Watanabe Y, Ozasa K, Higashi A, et al. Helicobacter pylori infection and atrophic gastritis. A case-control study in a rural town of Japan[J]*.* J Clin Gastroenterol, 1997; 25(1): 391-394.

5. Wang R,Chen XZ. Prevalence of atrophic gastritis in southwest China and predictive strength of serum gastrin-17: A cross-sectional study (SIGES)[J]*.* Sci Rep, 2020; 10(1): 4523.

6. Guarner J, Herrera-Goepfert R, Mohar A, et al. Diagnostic yield of gastric biopsy specimens when screening for preneoplastic lesions[J]*.* Hum Pathol, 2003; 34(1): 28-31.

7. Hosokawa O, Miyanaga T, Kaizaki Y, et al. Decreased death from gastric cancer by endoscopic screening: association with a population-based cancer registry[J]*.* Scand J Gastroenterol, 2008; 43(9): 1112-1115.

8. Yeh JM, Hur C, Kuntz KM, et al. Cost-effectiveness of treatment and endoscopic surveillance of precancerous lesions to prevent gastric cancer[J]*.* Cancer, 2010; 116(12): 2941-2953.

9. Hu J, Zhao Y, Ren M, et al. The Comparison between Endoscopic Submucosal Dissection and Surgery in Gastric Cancer: A Systematic Review and Meta-Analysis[J]*.* Gastroenterol Res Pract, 2018; 2018: 4378945.

10. Zhao Y,Wang C. Long-Term Clinical Efficacy and Perioperative Safety of Endoscopic Submucosal Dissection versus Endoscopic Mucosal Resection for Early Gastric Cancer: An Updated Meta-Analysis[J]*.* Biomed Res Int, 2018; 2018: 3152346.

11. Kondo A, de Moura EG, Bernardo WM, et al. Endoscopy vs surgery in the treatment of early gastric cancer: Systematic review[J]*.* World J Gastroenterol, 2015; 21(46): 13177-13187.

12. Rong L, Cai Y, Nian W, et al. Efficacy comparison between surgical resection and endoscopic submucosal dissection of early gastric cancer in a domestic single center[J]*.* Zhonghua Wei Chang Wai Ke Za Zhi, 2018; 21(2): 190-195.

13. Clinical guidelines for perioperative management of gastric endoscopic submucosal dissection[J]*.* Zhonghua Nei Ke Za Zhi, 2018; 57(2): 84-96.

14. Yang Z, Zeng H, Xia R, et al. Annual cost of illness of stomach and esophageal cancer patients in urban and rural areas in China: A multi-center study[J]*.* Chin J Cancer Res, 2018; 30(4): 439-448.

15. Xia R, Zeng H, Liu Q, et al. Health-related quality of life and health utility score of patients with gastric cancer: A multi-centre cross-sectional survey in China[J]*.* Eur J Cancer Care (Engl), 2020; 29(6): e13283.

16. Saumoy M, Schneider Y, Shen N, et al. Cost Effectiveness of Gastric Cancer Screening According to Race and Ethnicity[J]*.* Gastroenterology, 2018; 155(3): 648-660.

17. Vanni T, Karnon J, Madan J, et al. Calibrating models in economic evaluation: a seven-step approach[J]*.* Pharmacoeconomics, 2011; 29(1): 35-49.

18. You WC, Blot WJ, Li JY, et al. Precancerous gastric lesions in a population at high risk of stomach cancer[J]*.* Cancer Res, 1993; 53(6): 1317-1321.

19. Qin XX, Li WQ, Li ZX, et al. Trends of Gastric Cancer Incidence and Mortality from 2012 to 2019 in Linqu County, Shandong Province[J]*.* China cancer, 2021; 30(6): 415-421.

20. González CA, Pardo ML, Liso JM, et al. Gastric cancer occurrence in preneoplastic lesions: a long-term follow-up in a high-risk area in Spain[J]*.* Int J Cancer, 2010; 127(11): 2654-2660.

21. Valle J, Kekki M, Sipponen P, et al. Long-term course and consequences of Helicobacter pylori gastritis. Results of a 32-year follow-up study[J]*.* Scand J Gastroenterol, 1996; 31(6): 546-550.

22. Liu CY, Wu CY, Lin JT, et al. Multistate and multifactorial progression of gastric cancer: results from community-based mass screening for gastric cancer[J]*.* J Med Screen, 2006; 13 Suppl 1: S2-5.

23. Kuipers EJ, Uyterlinde AM, Peña AS, et al. Long-term sequelae of Helicobacter pylori gastritis[J]*.* Lancet, 1995; 345(8964): 1525-1528.

24. You WC, Li JY, Blot WJ, et al. Evolution of precancerous lesions in a rural Chinese population at high risk of gastric cancer[J]*.* Int J Cancer, 1999; 83(5): 615-619.

25. Plummer M, Vivas J, Lopez G, et al. Chemoprevention of precancerous gastric lesions with antioxidant vitamin supplementation: a randomized trial in a high-risk population[J]*.* J Natl Cancer Inst, 2007; 99(2): 137-146.

26. Leung WK, Lin SR, Ching JY, et al. Factors predicting progression of gastric intestinal metaplasia: results of a randomised trial on Helicobacter pylori eradication[J]*.* Gut, 2004; 53(9): 1244-1249.

27. Ma JL, Zhang L, Pan KF, et al. Helicobacter pylori and the progression of gastric cancer: a 10-year cohort study[J]*.* Zhonghua Yi Xue Za Zhi, 2005; 85(39): 2758-2761.

28. Correa P, Haenszel W, Cuello C, et al. Gastric precancerous process in a high risk population: cross-sectional studies[J]*.* Cancer Res, 1990; 50(15): 4731-4736.

29. den Hoed CM, Holster IL, Capelle LG, et al. Follow-up of premalignant lesions in patients at risk for progression to gastric cancer[J]*.* Endoscopy, 2013; 45(4): 249-256.

30. Chapelle N, Péron M, Quénéhervé L, et al. Long-Term Follow-up of Gastric Precancerous Lesions in a Low GC Incidence Area[J]*.* Clin Transl Gastroenterol, 2020; 11(12): e00237.

31. You WC, Brown LM, Zhang L, et al. Randomized double-blind factorial trial of three treatments to reduce the prevalence of precancerous gastric lesions[J]*.* J Natl Cancer Inst, 2006; 98(14): 974-983.

32. Correa P, Fontham ET, Bravo JC, et al. Chemoprevention of gastric dysplasia: randomized trial of antioxidant supplements and anti-helicobacter pylori therapy[J]*.* J Natl Cancer Inst, 2000; 92(23): 1881-1888.

33. Rugge M, Farinati F, Baffa R, et al. Gastric epithelial dysplasia in the natural history of gastric cancer: a multicenter prospective follow-up study. Interdisciplinary Group on Gastric Epithelial Dysplasia[J]*.* Gastroenterology, 1994; 107(5): 1288-1296.

34. Song H, Ekheden IG, Zheng Z, et al. Incidence of gastric cancer among patients with gastric precancerous lesions: observational cohort study in a low risk Western population[J]*.* Bmj, 2015; 351: h3867.

35. Kokkola A, Haapiainen R, Laxén F, et al. Risk of gastric carcinoma in patients with mucosal dysplasia associated with atrophic gastritis: a follow up study[J]*.* J Clin Pathol, 1996; 49(12): 979-984.

36. Tsukuma H, Oshima A, Narahara H, et al. Natural history of early gastric cancer: a non-concurrent, long term, follow up study[J]*.* Gut, 2000; 47(5): 618-621.

37. Iwai T, Yoshida M, Ono H, et al. Natural History of Early Gastric Cancer: a Case Report and Literature Review[J]*.* J Gastric Cancer, 2017; 17(1): 88-92.

38. Fujisaki J, Nakajima T, Hirasawa T, et al. Natural history of gastric cancer-a case followed up for eight years: early to advanced gastric cancer[J]*.* Clin J Gastroenterol, 2012; 5(5): 351-354.

39. Oh SY, Lee JH, Lee HJ, et al. Natural History of Gastric Cancer: Observational Study of Gastric Cancer Patients Not Treated During Follow-Up[J]*.* Ann Surg Oncol, 2019; 26(9): 2905-2911.

40. Bae JM, Shin SY,Kim EH. Mean sojourn time of preclinical gastric cancer in Korean men: a retrospective observational study[J]*.* J Prev Med Public Health, 2014; 47(4): 201-205.

41. González CA, Sanz-Anquela JM, Companioni O, et al. Incomplete type of intestinal metaplasia has the highest risk to progress to gastric cancer: results of the Spanish follow-up multicenter study[J]*.* J Gastroenterol Hepatol, 2016; 31(5): 953-958.

42. Adamu MA, Weck MN, Gao L, et al. Incidence of chronic atrophic gastritis: systematic review and meta-analysis of follow-up studies[J]*.* Eur J Epidemiol, 2010; 25(7): 439-448.

43. Kato I, Vivas J, Plummer M, et al. Environmental factors in Helicobacter pylori-related gastric precancerous lesions in Venezuela[J]*.* Cancer Epidemiol Biomarkers Prev, 2004; 13(3): 468-476.
